# Prevalence and Morphological Characteristics of the Femoral Head Ossification Nucleus in Chilean Infants: A Cross-Sectional Study

**DOI:** 10.3390/diagnostics15141814

**Published:** 2025-07-18

**Authors:** Marcelo Ortega-Silva, Mariano del Sol

**Affiliations:** 1 Programa de Doctorado en Ciencias Morfológicas, Facultad de Medicina, Universidad de La Frontera, Temuco 4780000, Chile; 2 Departamento de Ciencias Basicas, Facultad de Medicina, Universidad de La Frontera, Temuco 4780000, Chile; 3 Centro de Excelencia en Estudio Morfológicos y Quirúrgicos (CEMyQ), Facultad de Medicina, Universidad de La Frontera, Temuco 4780000, Chile

**Keywords:** developmental dysplasia, femoral ossification, radiographic screening, pediatric hip, orthopedics

## Abstract

**Background/Objectives:** Developmental dysplasia of the hip (DDH) affects 1–3% of newborns and requires early detection for optimal outcomes. DDH involves abnormal acetabular–femoral congruence between the acetabulum and femoral head, resulting from either shallow acetabular development or delayed femoral ossification of the femoral head. We evaluated the ossification nucleus of the femoral head (ONFH) to determine prevalence, radiographic timing, and associations with perinatal factors. **Methods:** We analyzed 100 pelvic radiographs of infants between 90 and 150 days of age. Participants were selected by convenience sampling, based on inclusion criteria. We identified the presence of ONFH and measured biometric parameters, morphology, and anatomical location. Sociodemographic and perinatal data were collected from the participating infants. **Results:** The prevalence of ONFH was 33%, and the mean age at visualization was 104 days. The presence of ONFH was correlated with birth weight (*p* = 0.011), discharge weight (*p* = 0.005), and weight at 1 month (*p* = 0.034). In our study, female sex (*p* = 0.004) was associated with a 4.966-fold higher odds of ONFH prevalence compared to males. **Conclusions:** This study provides relevant evidence regarding the prevalence, morphology, and characteristics of ONFH. Few studies report this information on ONFH in different populations. The optimal timing for radiographic visualization of ONFH in infants remains undefined, but the appearance of the ONFH was concentrated around 104 days of life. The novel association between weight and ONFH provides new insights into DDH. This provides new insights for DDH screening. This association warrants further research for the early detection of DDH.

## 1. Introduction

Developmental dysplasia of the hip (DDH) affects 1–3% of newborns, representing a significant pediatric orthopedic challenge [1]. Historically classified as congenital, the terminology has evolved to reflect both intrauterine and postnatal origins. Because diagnosis often occurs months postnatally, clinicians adopt ‘developmental’ to reflect origins both in utero and during hip [2]. Currently, DDH is recognized as a dynamic condition that may either progress or resolve over time [3]. Therefore, sequential clinical and imaging monitoring occurs at 2 weeks, then at 2, 4, 6, 9, and 12 months [4].

From an anatomical perspective, the hip is a synovial ball-and-socket joint characterized by congruence between the femoral head and the acetabulum. DDH disrupts this congruence through shallow acetabular development or abnormal femoral head positioning [5]. If these alterations are not detected in a timely manner, they can range from mild capsular laxity to early-onset osteoarthritis, functional impairments, or structural damage to the femur [2]. Advanced DDH causes chronic pain and permanent disability [6].

The main anatomical features of the pelvis develop during fetal life. During the first eight weeks of gestation, the acetabulum and femoral head form a mesenchymal block, which differentiates into a joint by approximately week 11. During this period, the cartilaginous femoral head begins to grow more rapidly than the acetabulum, a process that accelerates after birth [7]. At week 12, medial rotation of the lower limbs occurs, increasing the risk of dislocation. By week 18, the development of the hip musculature represents another critical phase, especially in the presence of neuromuscular disorders [8]. Finally, during the last four weeks of gestation, mechanical factors such as oligohydramnios or breech presentation may further increase the risk of dislocation; in the latter, dislocation incidence has been reported as high as 23% [9].

After birth, the acetabulum undergoes accelerated growth and improves femoral head coverage; however, the joint capsule remains lax, contributing to hip instability. During postnatal development, the proximal femoral epiphysis consists primarily of cartilage. Ossification begins at the appearance of a secondary center—the ossification nucleus of the femoral head (ONFH)—typically between 2 and 8 months of age. The timing of ONFH appearance remains debated in DDH [10]. In addition to its diagnostic relevance, there is ambiguity and scarcity of references in the literature regarding the prevalence and characteristics of the ONFH in pediatric populations. A delayed appearance of this nucleus has been reported in cases of unilateral or bilateral hip dislocation [11]. Early diagnosis during growth can significantly improve prognosis, whereas delayed diagnosis may lead to more severe pathologies [12]. This condition also entails high healthcare costs and negatively impacts the quality of life of patients and their social environments [13].

DDH can be diagnosed through clinical examination by a pediatric physician; however, imaging modalities are ultrasound and radiography, with their use primarily determined by the infant’s age [2]. Chilean guidelines require anteroposterior pelvic radiography for DDH screening [14]. Radiographic evaluation includes the assessment of pelvic bone maturation, among which the presence of the ONFH is a key parameter. ONFH appears radiographically in 20% of 3-month-old infants [15], and its absence suggests DDH risk [11]. This study aims to determine the prevalence, morphological variation, and timing of ONFH appearance in infants aged 90 to 150 days, as well as to evaluate its correlation with demographic and perinatal factors in a Chilean cohort.

## 2. Materials and Methods

### 2.1. Study Design and Sample

We conducted a cross-sectional study analyzing 100 pelvic radiographs from infants aged 90–150 days. Participants were selected by convenience sampling, based on inclusion criteria such as being within the target age range, undergoing their first screening radiograph, and having no diagnosis of DDH. All infants included in the study were residents of the commune of Temuco. Chilean guidelines recommend pelvic radiography at 3 months (90 days) for DDH screening [14]. The images used in this study corresponded to this routine clinical screening and did not involve any additional exposure to radiation. The sample consisted of 45 females (45%) and 55 males (55%) infants. Both hips were assessed in each radiograph to identify the presence of the ossification nucleus of the femoral head (ONFH) and to characterize its morphology, anatomical location, and spatial relationship with adjacent structures. We interviewed legal guardians for sociodemographic and perinatal data. Data sources included medical records and infant health records with guardian verification. Data sources include birth weight, discharge weight, weight at the first, second, and third months of life, gestational age, delivery mode, and feeding method.

### 2.2. Radiographic Technique

We obtained radiographs per national DDH guidelines [15] and positioned infants supine with extended lower limbs in parallel, symmetrical alignment, with gentle traction and anteriorly oriented patellae, preventing femoral rotation. The X-ray beam was centered at a standard distance of 100 cm, ensuring proper anatomical projection for the evaluation of hip joint development.

### 2.3. Inclusion Criteria for Radiographs

We included only radiographs meeting strict quality criteria. Which comprised centered imaging, bilateral hip symmetry, no pelvic rotation or tilt, no anteversion or retroversion, and symmetric obturator foramina and iliac wings. Additionally, we required clear visualization of the anatomy of the proximal femoral epiphyses, including both lesser trochanters, with diagnostic image quality and appropriate radiograph contrast.

### 2.4. Radiographic Assessment and Maternal Interview

We evaluated each image for ONFH presence. For visible nuclei, we performed biometric measurements, morphological analysis, laterality assessment, and anatomical localization. Concurrently, we interviewed legal guardians using medical records and health documentation to verify demographic and clinical data. This included exact age, sex, type of delivery, gestational age, type of feeding, birth weight, and weight at the first, second, and third months of life, and percentage of weight loss at hospital discharge.

### 2.5. Identification and Localization of the ONFH

**Figure 1:**
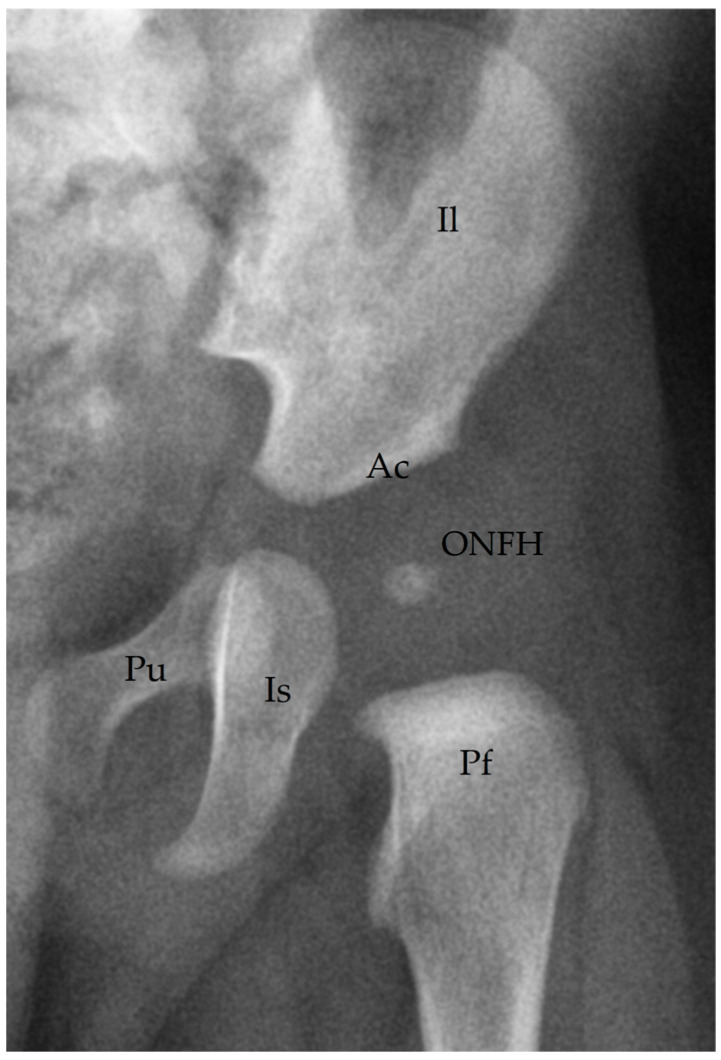
Anteroposterior radiograph of the pediatric pelvis. Il: Ilium; Ac: acetabulum; Pu: Pubis; Is: Ischium; Pf: Proximal Femur; ONFH: Ossification Nucleus of the Femoral Head.

We identified ONFH on anteroposterior radiographs (Figure 1). Anatomical landmarks included the proximal femoral epiphysis, acetabulum, pubis, and ischium. The ONFH appeared as a radiodense focus within the future femoral head.

### 2.6. Biometry of the Ossification Nucleus

**Figure 2:**
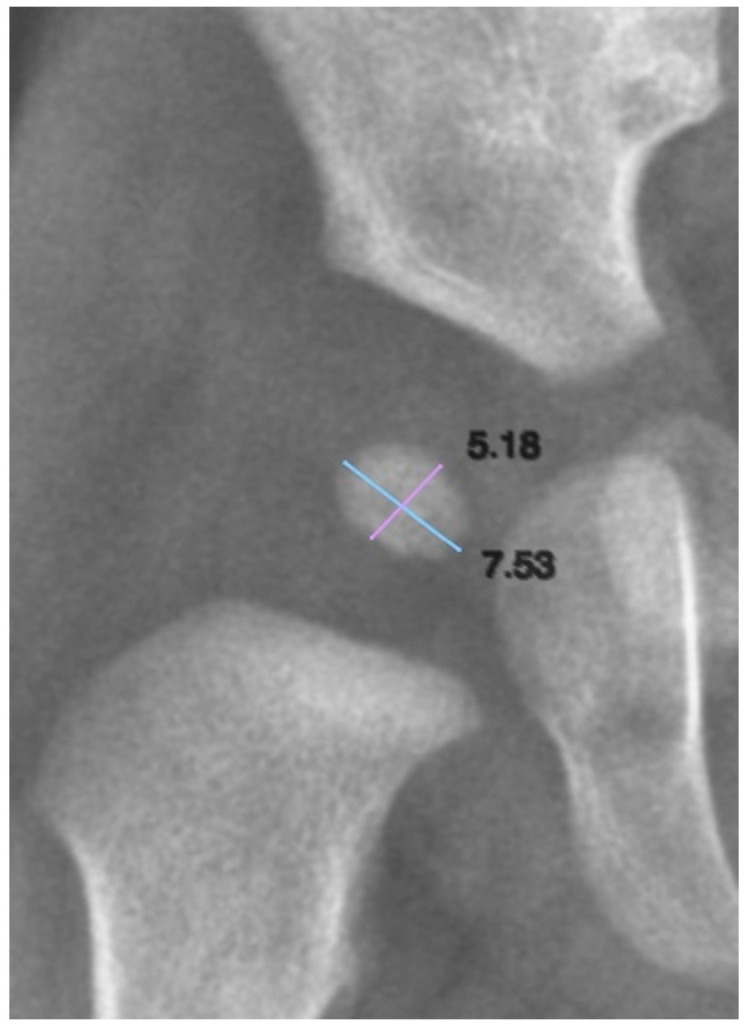
Biometric assessment of the ossification nucleus of the femoral head. Light blue line: transverse measurement axis; Purple line: longitudinal measurement axis.

To quantify the size of the ONFH, both its longitudinal and transverse axes were measured. We defined the longitudinal axis as the superoinferior dimension and the transverse axis as the mediolateral dimension (Figure 2). These measurements allowed for the assessment of nucleus growth and symmetry in relation to age and other clinical variables.

To assess the reliability of the measurements, the intraclass correlation coefficient (ICC) was calculated, allowing us to determine the strength of agreement for the biometric data.

Calibration used a phantom with known dimensions. A radiograph was obtained under standard image acquisition parameters with the phantom included, enabling pixel calibration in the imaging system based on the phantom’s known measurement.

### 2.7. Morphology of the ONFH

**Figure 3:**
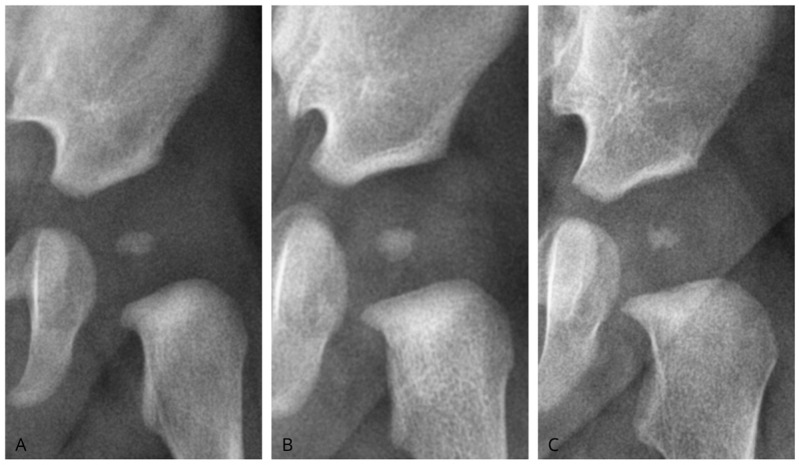
Morphology of the femoral head ossification nucleus. (**A**): Nucleus classified as oval; (**B**): Nucleus classified as round; (**C**): Nucleus classified as irregular.

We classified ONFH morphology as oval, round, or irregular (Figure 3). Oval nuclei had transverse dimensions exceeding longitudinal dimensions with homogeneous density. Round nuclei showed equal axes (±10%) with homogeneous density. Irregular nuclei displayed asymmetrical axes or uneven contours and heterogeneous density. To assess the reliability of the measurements, Cohen’s kappa coefficient was calculated to determine the strength of agreement among the different morphologies analyzed.

### 2.8. Positioning of the ONFH Relative to Anatomical Structures

**Figure 4:**
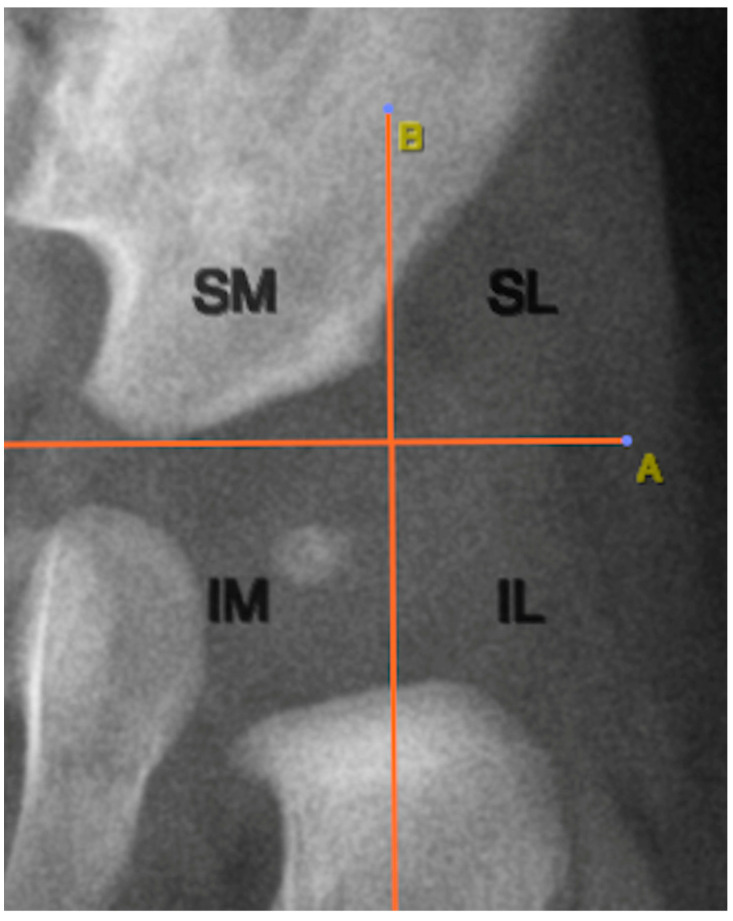
Quadrant-based localization of the ONFH relative to adjacent structures. A: Hilgenreiner’s line; B: Perkin’s lines; SM: superomedial quadrant; SL: superolateral quadrant; IL: inferolateral quadrant; IM: inferomedial quadrant.

The location of the ONFH was determined using two anatomical reference lines traced on the radiographic image. The first was Hilgenreiner’s line, a horizontal line connecting the lowest points of the triradiate cartilage of both iliac bones. Perkin’s lines extended perpendicular to Hilgenreiner’s line from the lateral acetabular edge. These lines created four quadrants: superolateral, superomedial, inferolateral, and inferomedial. Figure 4 illustrates this anatomical segmentation as applied to ONFH localization.

### 2.9. Statistical Analysis

Data collection was recorded using a Microsoft Office Excel spreadsheet. A descriptive analysis was performed, including the calculation of means, standard deviations, and frequency distribution tables. Normality was assessed using the Kolmogorov–Smirnov and Shapiro–Wilk tests. Pearson’s Chi-square test, independent samples t-test, one-way ANOVA, and multivariate analysis through binary logistic regression were also applied. Statistical analyses were conducted using SPSS Statistics for Windows (version 23.0, IBM Corporation, Armonk, NY, USA). A value *p* > 0.05 was considered the threshold for statistical significance.

## 3. Results

### 3.1. Prevalence and Laterality

ONFH was identified in 33% of the 100 infants included in the study. Among positive cases, 30 (91%) showed bilateral ossification. Only three cases presented unilateral ossification (one right, two left).

By sex, ONFH appeared in 20/45 females (44%) and 13/55 males (24%). Age showed no association with ONFH presence (*p* = 0.352), but sex was associated with ONFH presence, showing female predominance (*p* = 0.028).

The mean age of the infant at the time of the study was 104 days, with a range from 90 to 146 days. The distribution of ONFH presence across different age groups is detailed in Table 1.

### 3.2. Biometrics

ONFH measurements (longitudinal and transverse dimensions) appear in Table 2.

Reliability analysis showed ICC = 0.807 for length (good agreement) and 0.880 for width. Similarly, the ICC for the width measurements of the ONFH was 0.880, also reflecting good agreement. Statistical analysis of the ONFH length and width measurements in relation to age indicated that both variables were normally distributed. No differences existed between age groups for any dimension of the nucleus: right ONFH length (*p* = 0.662), right ONFH width (*p* = 0.885), left ONFH length (*p* = 0.773), and left ONFH width (*p* = 0.491). Sex comparisons revealed significant differences in width and form bilaterally (right: *p* = 0.043, left: *p* = 0.049). Males showed a greater width mean: 5.29 ± 1.17 mm vs. females: 5.32 ± 2.26 mm. For further detail, the individual biometric and morphological data of the ONFH for each subject are presented in Table S1 of the Supplementary Materials.

### 3.3. Morphology

Regarding ONFH morphology, the right hip presented an oval shape in 11 cases (35.5%), round in 17 cases (54.8%), and irregular in 3 cases (9.7%). In the left hip, 11 oval-shaped nuclei (34.4%), 16 round (50.0%), and 5 irregular (15.6%) cases were identified. Morphology showed no association with age (*p* = 0.127) or sex (*p* = 0.134). Similarly, no significant associations were observed between ONFH shape and sex on either the right side (*p* = 0.227) or the left side (*p* = 0.141), suggesting that ONFH morphology does not vary with these variables.

Cohen’s kappa coefficient was calculated to evaluate agreement between the described ONFH morphologies, yielding a kappa value of K = 0.444, which indicates a moderate level of agreement.

Analysis of ONFH localization revealed that all nuclei (100%) were located in the inferomedial quadrant, as defined by Hilgenreiner’s and Perkin’s lines (Figure 4).

### 3.4. Correlation with Demographic Factors

Regarding the type of feeding, 52% of the infants received exclusive breastfeeding (EBF), while 48% were formula-fed. The feeding method showed no association with age, sex, or ONFH presence. Likewise, no significant association was observed between feeding type and sex—EBF and sex (*p* = 0.334) or formula feeding and sex (*p* = 0.334). Furthermore, no statistical association was found between EBF and the prevalence of the ONFH (*p* = 0.179), nor between formula feeding and ONFH prevalence (*p* = 0.179).

With respect to the mode of delivery, 36% of births were vaginal and 64% were by cesarean section. As with feeding, no statistically significant associations were found between delivery mode and age (*p* = 0.119), sex (*p* = 0.451), or ONFH presence (*p* = 0.620).

Gestational age ranged from 36 to 42 weeks, with a mean of 38.4 ± 1.2 weeks. A statistically significant difference was found when comparing gestational age with the presence of the ONFH (*p* = 0.026), with the ossified infants having a higher mean gestational age of 38.8 weeks.

Regarding weight, data on birth weight, hospital discharge weight, and weight at the first, second, and third months of life are presented in Table 3. Significant differences existed between infants with and without ONFH for birth weight (*p* = 0.011), discharge weight (*p* = 0.005), and one-month weight (*p* = 0.034). Infants with ONFH showed higher mean weight: 3567 g at birth, 3345 g at discharge, and 4687 g at one month. However, no statistically significant differences were observed in weight at the second (*p* = 0.592) and third month of life (*p* = 0.757), nor in the percentage of weight loss at hospital discharge (*p* = 0.903).

The results of the logistic regression analysis are summarized below (Table 4). The reported statistics include the coefficients (B), standard errors, Wald’s statistics, significance values (*p*), odds ratio (Exp(B)), and 95% confidence intervals for each variable.

In our study, sex (1 = female, *p* = 0.004) was associated with a 4.966-fold higher odds of ONFH prevalence compared to males. Additionally, higher discharge weight (*p* = 0.001) was associated with a 1.003-fold increase in the odds of ONFH prevalence.

## 4. Discussion

Doberti, a Chilean radiologist, demonstrated that the cartilaginous femoral head critically influences acetabular development. Its absence should therefore be considered an early indicator in the diagnosis of developmental dysplasia of the hip (DDH) [16]. In Chile, the femoral head ossification nucleus (ONFH) is regarded as a key element in the radiological diagnosis of DDH. Radiologists consider it part of a diagnostic triad for this condition, which also includes lateral displacement of the distal femoral metaphysis and increased acetabular roof obliquity [14,15].

Given the limited literature on the prevalence of ONFH, the findings of this study will undoubtedly contribute to enriching the current body of knowledge on this topic, particularly by providing data from a different geographical region, such as Chile. Among the authors reviewed, Perez et al. (2023) reported that only 20% of infants presented ONFH ossification at three months of age, increasing to 80% by six months [15]; however, the population characteristics of the study were not specified. Another study reported a prevalence of 39% in a sample of 742 infants aged between 3 and 12 months, but again, no specific information on the study population was provided, and the broad age range raises concerns regarding the accuracy of prevalence estimation in comparison with our more narrowly defined cohort [17]. In our study population, we observed a 33% prevalence of ONFH in infants aged 90 to 150 days, allowing for a preliminary characterization of the Chilean pediatric population. This prevalence is particularly useful for estimating the expected frequency of ossification within this specific age range. However, the result should be interpreted with caution, as it represents only a sample of the Chilean population. We expect that the information obtained from this study will contribute to future research and support clinical decision-making, as standardizing prevalence benchmarks could help reduce unnecessary repetition of radiographic exams due to absent ONFH visualization—an absence that has been associated in the literature with potential DDH [11]. The repetition of radiological studies is a matter of concern, particularly in pediatric patients, given their greater sensitivity to ionizing radiation compared to adults [18]. Thus, having access to this data may also support the optimization of radiological protection protocols.

Regarding sex-related differences in the prevalence of this nucleus, authors such as Gutiérrez et al. (1999) have reported an earlier appearance of the ONFH in females [11]. Similarly, Cobos et al. (2023) describe a faster pattern of bone maturation in girls, including earlier epiphyseal fusion [19] and more efficient ossification in both primary and secondary ossification centers, compared to males [20]. Our findings are consistent with these observations, as we found a higher prevalence of ONFH in female infants, a difference that was statistically significant. We believe this finding is primarily attributable to earlier skeletal maturation in females compared to males, a process likely mediated by hormonal factors [21]. Therefore, future research should investigate the hormonal profiles of both sexes in order to better elucidate their role in the timing and progression of ossification. It is important to highlight that the binary logistic regression yielded an odds ratio of 4.966 for sex in relation to ONFH prevalence, which further supports the findings previously described by other authors and provides substantial evidence within our population.

In terms of laterality, the data showed an even distribution between the right and left hips. However, based on the analysis of bilaterality in our sample (91%), we note that this finding is noteworthy in the context of existing literature, as none of the consulted authors explicitly addressed this aspect. We consider this point important, given that a delayed appearance of the ONFH on one side has been reported to be associated with the presence of DDH [22]. Knowing that the ONFH appears bilaterally in a high percentage of infants would allow for the establishment of normative patterns, aligning with previous observations that unilateral presentation may indicate a pathological condition. For future DDH studies, this parameter should be considered in order to achieve a more comprehensive evaluation.

The age range of the subjects was 90 to 146 days. The analysis showed that the average age at which the ONFH was observed was 104 days, which contrasts with the ministerial recommendation of performing radiography at three months of age. This finding highlights the need to reassess the optimal timing for conducting this examination in the Chilean population, as our results suggest that performing the radiograph at 104 days may increase the likelihood of identifying the ONFH and help avoid repeat imaging, with clear benefits for the patient, the healthcare system, and the family. This finding should undoubtedly be further evaluated in additional studies to ensure its proper clinical application and should be considered in multicenter research efforts to provide a stronger theoretical foundation.

In 15-day age groups, ONFH frequency peaked in the earliest group. However, this difference was not statistically significant, which may be due to a higher number of infants in that specific age bracket.

ONFH measurements showed transverse exceeded longitudinal dimensions, averaging 5.15 mm (right) and 5.28 mm (left). This predominance results in an overall oval morphology and represents a novel contribution to the literature, as no previous biometric data for the ONFH in this age group have been reported. These measurements may prove useful in the differential diagnosis of conditions affecting the femoral head, such as avascular necrosis or DDH. No significant differences in size were observed based on age or sex, although it is noteworthy that the smallest dimensions were recorded in female infants.

Morphologically, we identified three shapes: irregular, round, and oval. Irregular form predominated in the early stages of ossification, whereas round and subsequently oval forms appeared with increasing age. Despite non-significant associations between morphology and age or sex, longitudinal studies could explore developmental patterns. Such research could aim to establish a morphogenetic sequence of the ONFH linked to chronological age.

Among the imaging analyses specific to pediatric pelvic radiography, the positioning of the ONFH is of particular interest. In our study, all cases of ONFH were located in the inferomedial quadrant of the hip joint, as defined by Hilgenreiner’s and Perkin’s lines, suggesting that this represents their normal physiological location. Therefore, the presence of the ONFH outside this quadrant could be indicative of hip dislocation [15].

With respect to clinical factors, no association was found between the type of feeding (exclusive breastfeeding or formula feeding) and the presence of the ONFH. However, we believe that this variable merits further investigation, considering not only the infant’s feeding method but also maternal nutrition during pregnancy, which could influence fetal skeletal development.

Similarly, no relationship was observed between the type of delivery and the presence of the ONFH. While previous studies have suggested that vaginal delivery may increase the risk of DDH compared to cesarean section [23], our data did not show a statistically significant association with the presence of the ossification nucleus. Nonetheless, this does not rule out a potential relationship with other clinical manifestations of DDH.

Regarding gestational age, a significant difference was observed: infants presenting with the ONFH had a longer average gestational period (38.8 weeks). This finding suggests that greater intrauterine development may promote the appearance of the ossification nucleus. However, it is well established that prolonged gestation increases the risk of DDH due to mechanical constraints within the uterus [5].

Finally, birth weight, discharge weight, and weight at one month of age were all significantly associated with the presence of ONFH. Infants who presented ossification had higher weights at these time points, suggesting that greater early weight gain may favor the appearance of the ONFH. This association was not sustained at two and three months of age. This novel finding warrants further investigation. As it has not been previously reported, its potential relationship with ONFH development may position it as a risk factor, making it a valuable area for further research. This is further supported by the binary logistic regression analysis, which revealed a statistically significant association between discharge weight and ONFH prevalence, with an odds ratio of 1.003. Therefore, we emphasize the importance of considering weight—particularly this variable— in future studies.

As limitations of this study, being observational in nature, we cannot establish causal relationships between the variables analyzed, which may represent risk factors in our population. Therefore, we recognize the need to conduct multicenter studies to better validate these new findings, aiming to enhance scientific rigor by employing an experimental design.

## 5. Conclusions

This study provides relevant evidence regarding the prevalence, morphology, and characteristics of the ossification nucleus of the femoral head (ONFH) in Chilean infants aged 90 to 150 days. A prevalence of 33% was identified, with bilateral ossification and inferomedial localization as the predominant and likely physiological pattern. Biometric parameters of the ONFH were established, and its most frequent morphological types were described, with the oval shape being the most representative of advanced ossification stages.

One of the most relevant findings was that the appearance of the ONFH was concentrated around 104 days of life, suggesting that this may represent the optimal timing for performing pediatric pelvic radiography in this population, potentially improving diagnostic efficiency and reducing the need for repeat imaging. This observation highlights the importance of conducting future studies on this variable, with the aim of standardizing local practices through multicenter research and supporting its potential clinical application. Additionally, associated clinical variables were identified, including female sex, higher gestational age, greater birth weight, and higher weight at one month of age. These factors may serve as guiding clinical criteria when planning imaging evaluations.

Binary logistic regression enabled the identification of variables significantly associated with the prevalence of the condition under study. In this case, “sex” and “discharge weight” showed statistically significant associations according to the Wald criterion. It is recommended that these factors be considered in clinical follow-ups.

These findings make a substantive contribution to the understanding of femoral head ossification in the context of early DDH diagnosis and open new avenues for future clinical and morphological research.

## Figures and Tables

**Table 1:** Presence of the ossification nucleus of the femoral head (ONFH) by age group.

Age Group (Days)	ONFH Present	ONFH Absent	ONFH Prevalence (%)	Cumulative ONFH Prevalence (%)	Confidence Interval—95% CI	Total
90–105 days	18	41	54.54	54.54	[94.47; 96.79]	59
106–120 days	12	19	36.36	90.9	[109.82; 112.89]	31
121–135 days	1	6	3.03	93.93	[122.44; 131.84]	7
136–150 days	2	1	6.07	100	[129.62; 152.38]	3

**Table 2:** Descriptive statistics of ONFH length and width measurements.

Variable	Minimum (mm)	Maximum (mm)	Mean (mm)	Standard Deviation (mm)
Right Length	1.83	7.14	4.39	1.38
Right Width	2.24	9.34	5.15	1.81
Left Length	1.40	7.45	4.43	1.42
Left Width	1.09	9.60	5.28	1.96

**Table 3:** Descriptive statistics for weight, discharge weight, weight at the first, second, and third months of life, and percentage of weight loss at discharge.

Variable	Minimum (g)	Maximum (g)	Mean (g)	Standard Deviation
Birth weight	2305	4290	3418	414
Hospital discharge weight	2100	3945	3188	394
Weight at the first month of life	2940	6100	4486	649
Weight at the second month of life	3685	7250	5505	677
Weight at the third month of life	4865	8300	6325	713
% Weight loss at hospital discharge *	1.6%	10.7%	6.9%	1.8%

* Ratio between birth weight and hospital discharge weight.

**Table 4:** Binary Logistic regression.

Variable	B	Standard Error	Wald	Sig.	Exp(B)
Sex	1.603	0.558	8.241	0.004	4.966
Discharge Weight	0.003	0.001	10.537	0.001	1.003

## Data Availability

The data supporting the findings of this study are available from the corresponding author upon reasonable request. However, part of the dataset is derived from clinical records and is subject to legal and ethical data protection regulations.

## Supplementary Materials

The following supporting information can be downloaded at: https://www.mdpi.com/article/10.3390/diagnostics15141814/s1, Table S1: Biometric and Morphological data by subject.
