# Supplementary material for: Prevalence and Morphological Characteristics of the Femoral Head Ossification Nucleus in Chilean Infants: A Cross-Sectional Study
Source: Diagnostics (Basel). 2025 Jul 18;15(14):1814. doi: 10.3390/diagnostics15141814 (PMC12293775; doi:10.3390/diagnostics15141814)
Supplement: Supplementary file 1 [file diagnostics-15-01814-s001.zip › diagnostics-3725264-supplementary.pdf]

Prevalence and Morphological Characteristics of the Femoral Head Ossification Nucleus in Chilean Infants: A Cross-Sectional Study

Marcelo Ortega-Silva [1,2,3,*] and Mariano del Sol [1,3,*]

[1] Programa de Doctorado en Ciencias Morfológicas, Facultad de Medicina, Universidad de La Frontera, Temuco 4780000, Chile
[2] Departamento de Ciencias Basicas, Facultad de Medicina, Universidad de La Frontera, Temuco 4780000, Chile
[3] Centro de Excelencia en Estudio Morfológicos y Quirúrgicos (CEMyQ), Facultad de Medicina, Universidad de La Frontera, Temuco 4780000, Chile
* Correspondence: marcelo.ortega@ufrontera.cl (M.O.-S.); mariano.delsol@ufrontera.cl (M.d.S.)

Supplementary Material

Table S1: Biometric and morphological data by subject.

ID	Right ONFH-Length (mm)	Right ONFH-Width (mm)	Left ONFH-Length (mm)	Left ONFH-Width (mm)	Right ONFH morphology	Left ONFH morphology
2	5,75	4,66	7,45	5,28	Round	Round
5	6,17	2,67	4,79	4,06	Oval	Round
7	7,14	4,25	5,9	3,77	Oval	Oval
11	3,59	2,69	1,4	1,09	Irregular	Irregular
12	6,45	8,2	6,45	8,29	Round	Round
16	4,76	5,88	4,35	6,26	Round	Round
19	3,92	4,2	3,92	4,91	Round	Round
23	2,8	5,32	4,2	6,02	Oval	Oval
25	6,19	5,35	6,81	5,62	Round	Round
27	5,19	4,76	5,54	4,42	Round	Oval
28	2,42	2,24	-	-	Round	-
33	3,36	4,34	4,2	5,18	Round	Round
39	2,81	3,96	2,8	5,05	Round	Oval
44	-	-	1,96	1,54	-	Round
53	1,83	2,38	3,08	2,94	Round	Round
61	3,78	4,62	3,78	6,17	Round	Oval
64	-	-	3,92	5,19	-	Round
67	4,36	5,99	4,62	6,44	Oval	Round
68	4,2	5,07	3,87	6,25	Round	Oval
70	3,8	3,6	3,5	3,22	Round	Round
73	5,01	7,08	5,89	8,24	Oval	Oval

75	4,76	6,38	4,35	3,96	Round	Irregular
77	6,21	9,34	5,64	9,17	Oval	Oval
79	4,29	7,11	4,7	6,49	Oval	Oval
81	6,69	9,09	6,89	9,6	Oval	Oval
83	3,48	4,85	3,59	5,02	Oval	Round
84	3,36	4,06	4,06	4,62	Round	Round
86	3,72	3,67	2,35	3,13	Irregular	Irregular
90	3,37	4,92	3,64	5,54	Oval	Oval
95	3,46	5,6	4,36	5,07	Oval	Round
96	3,64	4,65	3,94	3,36	Irregular	Irregular
99	3,39	5,01	4,06	5,59	Round	Irregular
100	6,14	7,7	5,89	7,33	Round	Round
